# Profile of Acute Kidney Injury in the Pediatric Age Group in a Tertiary Care Hospital: A Prospective Observational Study

**DOI:** 10.7759/cureus.54236

**Published:** 2024-02-15

**Authors:** Ishan Kapil, Anil Kumar Goel, Manas R Sahoo

**Affiliations:** 1 Paediatrics, All India Institute of Medical Science, Raipur, Raipur, IND; 2 Paediatrics, All India Institute of Medical Sciences, Raipur, Raipur, IND; 3 Pediatrics, All India Institute of Medical Sciences, Raipur, Raipur, IND

**Keywords:** peritoneal dialysis (pd), chronic kidney disease (ckd), renal replacement therapy, intensive care unit, acute kidney injury

## Abstract

Background and objectives: Acute kidney injury (AKI) is a menace in the pediatric intensive care unit (PICU) and is responsible for significant morbidity and mortality all over the world. There are limited data available on pediatric AKI in central India. Our primary objective is to determine the clinical, etiological, and outcome profile of AKI in the pediatric age group of 3 months to 15 years admitted to the All India Institute of Medical Sciences (AIIMS), Raipur. The secondary objective(s) is to predict the association of mortality in children diagnosed with AKI and to estimate the number of patients developing chronic kidney disease (CKD) at three-month follow-up.

Materials and methods: This observational study was conducted in the Department of Pediatrics at AIIMS Raipur, Chhattisgarh, from September 2021 to February 2023. All patients aged 3 months to 15 years of age satisfying the Kidney Disease: Improving Global Outcomes (KDIGO) criteria for AKI and presenting to the hospital were included, and those refusing consent or having CKD stage ≥3 were excluded. A total of 66 children were assessed for eligibility. Out of these 66 patients, 2 were excluded as they had AKI on CKD, and a total of 64 patients were included. For all included patients, details of their demography, clinical features, etiology workup, and hospital stay were collected. Their outcome was observed and categorized into complete response, partial response, no response, left against medical advice (LAMA), or death. Patients who were discharged were followed up for three months and observed for the recovery or development of CKD.

Results: The incidence of AKI in the PICU was 15.48% (64/413). Ventricular septal defect with pneumonia and pneumonia (12.5%, 8/64 each) were the most common diagnoses at presentation, resulting in AKI. The most common clinical presentations were fever (54.7%, 35/64) and respiratory distress (43.8%, 28/64). Out of them, 73.4% (47/64) had sepsis, and 62.5% (40/64) had shock. About 56.2% (36/64) of children had non-oliguric AKI as compared to 43.8% (28/64) who had oliguric AKI. Among total children with AKI, 54.7% (35/64) of patients had prerenal AKI, 43.8% (28/64) had renal AKI, and 1.6% (1/64) had postrenal AKI. Of all the children included, 32.8% (21/64) experienced complete resolution of AKI, while 18.8% (12/64) showed partial resolution, and 1.6% (1/64) remained unresolved. Among them, 3.1% (2/64) LAMA, and 43.8% (28/64) died. The median duration of the hospital stay in our study was 16.5 days. Out of them, 59.4% (38/64) of patients required renal replacement therapy (60.5% required peritoneal dialysis (PD), 36.8% required hemodialysis (HD), and 2.6% required both). Among survivors, 19.35% (6/31) developed CKD on a three-month follow-up.

Conclusion: The incidence of AKI was seen in critically ill children in the PICU, and it was associated with high mortality.

## Introduction

A sudden decline in the functioning of the kidney is referred to as an acute kidney injury (AKI). It is a broad clinical term that encompasses a variety of etiologies, such as extrarenal pathology (pre-renal azotemia and acute postrenal obstructive nephropathy) and some kidney diseases (such as acute interstitial nephritis and acute glomerular and vascular renal diseases). AKI is characterized by an increase in serum creatinine of ≥0.3 mg/dl within 48 hours, a rise to 1.5 times baseline serum creatinine that happened within the previous seven days, or a drop in urine output to ≤0.5 ml/kg/h for six hours [1]. The overall incidence of AKI was around 25% in children admitted to wards and pediatric intensive care units (PICUs) in various Indian studies [2,3]. The etiology of AKI in developed nations is mostly secondary to surgeries and the use of nephrotoxic drugs [4,5], whereas the most common etiologies in India, as seen from various studies, are infections, diarrhea with dehydration, and sepsis [6-8]. According to worldwide epidemiological data, even mild, reversible AKI has significant clinical repercussions, such as a higher risk of death [9,10].

In this study, we identified the demographic, clinical, etiological, and outcome profiles of pediatric patients between the age groups of 3 months and 15 years diagnosed with AKI in a tertiary care hospital in central India and predicted the association of AKI with mortality.

## Materials and methods

Study design and setting

This prospective observational study was conducted in the Department of Pediatrics at AIIMS Raipur from September 2021 to February 2023. All patients aged 3 months to 15 years of age satisfying the Kidney Disease Improving Global Outcomes (KDIGO) criteria of AKI and presenting to the PICU were included, and those refusing consent or having chronic kidney disease (CKD) stage ≥3 were excluded. Consent was obtained from the parents before enrolling in the study. For all included patients, details of their demography, clinical features, etiology workup, and hospital stay were collected. Age groups were divided into 3 months to 12 months, 1-5 years, 6-10 years, and 11-15 years. The maximum stage of AKI attained during the hospital stay was taken to be the stage of AKI in a particular patient. Their outcome was observed and categorized into complete recovery, partial recovery, no recovery, left against medical advice (LAMA), or death. Complete recovery was described as the absence of AKI on discharge; partial recovery was defined as persistent AKI with a decrease in AKI stage at discharge when compared with the maximum stage of AKI during admission; and no recovery was the persistence of maximum stage AKI or worsening of AKI on discharge. Patients who were discharged were followed up for three months, and the development of CKD was observed. The study was approved by the institute ethics committee with approval number AIIMSRPR/IEC/2021/941.

The sample size was calculated using the formula for observational studies (4pq/d2), after which the sample size came out to be 55, taking into consideration the study conducted by Krishnamurthy et al. [2].

(4 x 16.66 x 83.34)/10 x 10 = 55

55 + lost to follow-up 20% = 65

[p = proportion of commonest etiology (pneumonia) of AKI = 16.66%]

[q = (1-p) = 83.34%]

[d = absolute precision = 10%]

Data analysis

All data were recorded on a predesigned case sheet after obtaining consent from the participants. The data were compiled in Microsoft Excel (2019) (Microsoft® Corp., Redmond, WA). SPSS v23 (IBM Corp., Armonk, NY) was used for statistical analysis. We used means, standard deviations, and medians to describe continuous data, and frequencies and percentages to describe categorical variables. Wherever possible, data were displayed graphically for visualization using histograms, box-and-whisker plots, column charts, and pie charts for categorical data, and bar charts for continuous data. When comparing two groups, we used the independent sample 't' test to make group comparisons for continuously distributed data. The normality of the data was checked by using the Shapiro-Wilk test. If the data were found to be not normally distributed and not paired, Mann-Whitney U tests were performed. For categorical data group comparisons, the chi-squared test was employed. The Fisher's exact test was employed in place of the contingency tables if the anticipated frequency was discovered to be <5 for >20% of the cells. Both Pearson's correlation and Spearman's correlation were used to examine the linear correlation between two continuous variables, depending on whether the data were regularly distributed or not. The data were considered to be statistically significant at p < 0.05. Univariate and multivariate logistic regression were used as predictors of mortality.

## Results

During the study period, total admissions to the PICU were 414. Eligibility criteria were met by 66 patients, but 2 were excluded because of AKI on CKD. So, the incidence of AKI in the PICU was 15.48% (64/414). Our study included 33 boys and 31 girls; all age groups were almost equally represented, but the 6-10-year age group constituted the maximum percentage, 28.1% (18/64). Ventricular septal defect (VSD) with pneumonia and pneumonia (12.5%, 8/64 each) were the most common diagnoses at presentation, resulting in AKI (Table 1). The most common clinical presentations were fever, 54.7% (35/64), and respiratory distress, 43.8% (28/64). Out of the total cases, 73.4% (47/64) had sepsis, and 62.5% (40/64) had shock. The most common electrolyte abnormalities were hyponatremia (42.18%, N=27), hyperkalemia (37.5%, N=24), and 90.6% (N=58) patients had high anion gap metabolic acidosis. Non-oliguric AKI was seen in 56.2% (36/64) of children, as compared to 43.8% (28/64) who had oliguric AKI. Prerenal AKI was seen in 54.7% (35/64) of patients, while 43.8% (28/64) had renal AKI and 1.6% (1.64) had postrenal AKI. Out of the total of the participants, 10.9% (7/64) were categorized as having stage 1 AKI, 7.8% (5/64) as stage 2, and 81.2% (52/64) constituting the maximum percentage, having stage 3 AKI as per the KDIGO classification. Out of all children included, 32.8% had completely resolved AKI, 18.8% had a partial resolution, 1.6% had unresolved, 3.1% left against medical advice, and 43.8% died. The median duration of the hospital stay in our study was 16.5 days (IQR 9 to 30.25) (Table 1). The mean pSOFA score was 6.7, whereas the mean PRISM3 score was 10.2. There was a statistically significant association between mortality and a higher PRISM3 score with a p-value < 0.01 (Table 2). However, on multivariate analysis, no significance was noted (Table 3). About 59.4% (N=38) of patients required renal replacement therapy (60.5% required PD, 36.8% required hemodialysis (HD), and 2.6% required both). Out of those who survived, 19.35% (6/31) developed CKD at the three-month follow-up.

**Table 1:** Etiology of acute kidney injury in study population

Diagnosis	Frequency (N=64) percentage
Pneumonia	8(12.50%)
Acyanotic heart disease	8(12.50%)
Nephrotic syndrome	4(6.30%)
Acute gastroenteritis	4(6.30%)
Systemic lupus erythematosus	3(4.70%)
Sickle cell disease	3(4.70%)
Hemolytic uremic syndrome	3(4.70%)
Encephalitis	3(4.70%)
Acute lymphoblastic leukemia	3(4.70%)
Meningitis	2(3.10%)
Lymphoma	2(3.10%)
Diabetic ketoacidosis	2(3.10%)
Cellulitis	2(3.10%)
Status epilepticus	1(1.60%)
Scorpion envenomation	1(1.60%)
Rapidly progressive glomerulonephritis	1(1.60%)
Pyelonephritis	1(1.60%)
Posterior urethral valve	1(1.60%)
Post streptococcal glomerulonephritis	1(1.60%)
Neurotoxic snakebite	1(1.60%)
Miliary tuberculosis	1(1.60%)

**Table 2:** Predictors of mortality on univariate analysis HAGMA: high anion gap metabolic acidosis, eGFR: estimated glomerular filtration rate, PRISM3 score: Pediatric Risk of Mortality 3 score

Dependent: mortality		Yes	OR	P-value	95% CI
AKI type	Prerenal	17 (48.6)			
	Renal	11 (39.3)	0.69	0.462	0.25–1.88
	Postrenal	0 (0.0)	0.00	0.991	0.00–Inf
Stage	Stage 1	3 (42.9)	-		
	Stage 2	3 (60.0)	2.00	0.560	0.19–20.6
	Stage 3	22 (42.3)	0.98	0.978	0.20–4.82
Shock	Yes	17 (42.5)	0.87	0.795	0.32–2.42
Renal replacement therapy	Yes	18 (47.4)	1.44	0.481	0.52–3.97
Pneumonia	Yes	6 (75.0)	4.64	0.075	0.86–25.0
Acyanotic heart disease (VSD)	Yes	3 (37.5)	0.74	0.704	0.16–3.42
Age	3–12 months	8 (50.0)	-		
	1–5 years	6 (40.0)	0.67	0.577	0.16–2.77
	6–10 years	5 (27.8)	0.38	0.188	0.09–1.60
	11–15 years	9 (60.0)	1.50	0.577	0.36–6.23
Gender	Male	16 (51.6)	-		
	Female	12 (36.4)	0.54	0.221	0.20–1.46
Vomiting, loose stool	Yes	5 (23.8)	0.27	0.029	0.08–0.88
Decreased UO	Yes	2 (15.4)	0.17	0.033	0.04–0.87
Hyponatremia	Yes	9 (33.3)	1.20	0.344	0.28–2.94
HAGMA	Yes	26 (44.8)	1.34	0.148	0.08–4.8
eGFR	≥35 mL/1.73 m²	21 (55.3)	-		
	<35 mL/1.73 m²	7 (26.9)	0.30	0.028	0.10–0.88
PRISM3 score	Mean (SD)	11.6 (3.5)	1.14	0.047	1.00–1.29
Ventilation requirements	Yes	22 (55.0)	3.67	0.022	1.20–11.1

**Table 3:** Predictors of mortality on multivariate analysis PRISM3 score: Pediatric Risk of Mortality score 3

Dependent: mortality		Yes	OR (multivariable)	P-value	95% CI
PRISM3 score	Mean (SD)	11.6 (3.5)	1.10	0.168	0.96–1.25
Ventilation requirements	Yes	22 (55.0)	2.56	0.120	0.78–8.3

## Discussion

The incidence of AKI in critically ill children in our PICU unit was 15.48%. In a meta-analysis done by Meena et al., published in 2023 by the American Academy of Pediatrics, the worldwide incidence of AKI was 26% in pediatric intensive care units [11]. Incidence in critically ill children was 9.8% in a study done by Rao et al. in Raipur, Chhattisgarh [12]. Another study done in North India in 2012 by Mehta et al. showed an incidence of 15% of AKI in admitted children and 36.1% in critically ill children [13]. Our study was done during the COVID pandemic, due to which we could not admit many patients. This could be an explanation for such an incidence.

The single most common diagnosis at presentation was VSD with pneumonia and pneumonia (12.5 % each, N = 8 each). The most common clinical presentations were fever (54.7%, N = 35) and respiratory distress (43.8%, N = 28). In patients with VSD, seven out of eight patients had pneumonia (87.5%), which led to pneumonia being the most common etiology. Other causes of AKI included four cases of nephrotic syndrome, three cases of HUS, one each of post-streptococcal glomerulonephritis, rapidly progressive glomerulonephritis, posterior urethral valve, pyelonephritis, and complicated UTI. Being a tertiary care center, we got referrals for respiratory illnesses and cardiac cases for surgery, which might explain the types of cases in our study. In a study done by Ashish et al. published in 2023 from a tertiary hospital in North India, diarrhea with dehydration was the most common cause of AKI (39%) [6]. From South India, Murdeshwar et al. also published their study in 2023, which showed 34.5% of children having acute glomerulonephritis as the most common cause of acute kidney disease [8]. As per Rao et al., who did their study in central India, the maximum number of patients diagnosed with new-onset AKI had primary CNS involvement (26.2%), with infections being the most common cause overall (9.2%), with pneumonia taking the top spot [12]. Kumar et al. did a cross-sectional study in 2019 comparing the etiologies of AKI in two centers, one in Uttarakhand and the other in Maharashtra. The hospital in Uttarakhand reported dehydration (69.3%) to be the most common cause of AKI, whereas the center in Maharashtra reported sepsis with MODS to be the most common cause [7].

Non-oliguric AKI was seen in 56.2% (N = 36), and 43.8% (N = 28) had oliguric AKI. In a study done by Krishnamurthy et al., 44.44% of patients had oliguria or anuria in the PICU, while in two observational studies from Pakistan by Bai et al. and Tresa et al., fever (78.5% vs. 65.5%) and oliguria or anuria (53.1% vs. 83.6%) were common complaints in children with AKI [2,14,15].

AKI was prerenal in 35 (54.68%), renal in 28 (43.75%), and postrenal in a single (1.5%) patient. In the Krishnamurthy et al. study, the distribution of prerenal, renal, and postrenal AKI was 24.09%, 74.09%, and 1.8%, respectively, and it was 50.76%, 40.7%, and 8.4% in the study from Pakistan by Bai et al. [2,14]. Here, our result was comparable to the latter study because 62.5% of the patients in our study had shock.

Causes like diarrhea were not common at our center (6.2%, N=4), probably because it is a tertiary referral center and most of such cases get treated at the local level. Seven out of eight patients with pneumonia were ventilated. Hence, pneumonia was one of the most important causes in our study, which is in agreement with results from other studies as quoted earlier. Causes like VSD, which are not able to be treated at the local level and thus referred to, constituted a significant number (N=8), and seven out of eight (87.5%) were associated with pneumonia.

In our study, we found that 62.5% of patients had shock during their hospital stay, 73.4% of patients had sepsis, and 62.5% of children required mechanical ventilation. The most common cause of death among VSD and pneumonia was catecholamine-refractory septic shock. Out of eight pneumonia cases, 75% (N=6) expired, contributing the most to mortality among the varying etiologies. The requirement of ventilation and PRISM3 score were independent predictors of mortality, with the odds ratios being 3.67 (95% CI = 1.00-1.29) and 1.14 (95% CI = 1.20-11.1), respectively (p < 0.05). In a study by Rakhmavati et al. on predictors of mortality in children with AKI, severe AKI (defined as having injury, failure, loss, or end-stage as per pRIFLE criteria), ventilator use, and infections were independent predictors of the same [16]. The study by Krishnamurthy et al. found that meningoencephalitis, hyponatremia, and hypernatremia were independent predictors of mortality [2].

The median duration of the hospital stay in our study was 16.5 days (IQR 9-30.25). The mean duration of hospital stay in patients with AKI completely recovering and in patients dying was 37.17 and 15.43 days, respectively (p-value = 0.016). This could be a consequence of more critically ill patients dying earlier and patients with milder illnesses surviving but requiring longer hospital stays. The median duration of hospital stay increased in patients in different stages of AKI: 6, 10, and 19.5 days in KDIGO stages 1, 2, and 3, respectively. A similar result was obtained in a study done by Ashish et al., where median durations were 4, 5.5, and 6 days in stages 1, 2, and 3, respectively [6].

In our study, most patients were classified into stage 3 AKI (81%; N=52), followed by stage 1 AKI (11%; N=7) and stage 2 AKI (8%; N=5). In the study by Mehta et al. on hospitalized patients from Delhi in 2012, children belonging to stage 1 were 48 (65.75%), stage 2 were 13 (17.80%), and stage 3 were 16.43% [13]. Krishnamurthy et al. [2] found stages 1, 2, and 3 in 36.74%, 34.93%, and 28.31% of patients, respectively, in South India. Hence, we see that most patients coming to our center had stage 3 AKI, which is unlike other studies done.

The majority of patients belonged to stage 3 AKI (N=52), 42.3% (N=22), succumbed, as compared to 42.9% (N=3) and 60% (N=3), dying in stages 1 and 2, respectively. The number of patients in stages 1 (N=7) and 2 (N=5) was significantly less than that in stage 3 (N=52). Patients in stage 3 AKI received RRT on time, which prevented their deaths, and received prolonged care in the hospital. Patients in stages 1 and 2 died as a result of some other illness by the time their AKI was diagnosed. This is supported by the fact that 71% (N=5) and 80% (N=4) of patients in stages 1 and 2 required ventilation, as compared to 59.6% (N=31) of patients in stage 3 AKI. The stagewise mean PRISM3 score was 8.71, 8.6, and 10.56 in stages 1, 2, and 3, respectively. In stages 1, 2, and 3, the percentage of patients who survived was 57.1%, 40%, and 57.69%, respectively.

Out of those in stage 3 (N=52), 59.4% required dialysis. Complete resolution was seen in 30.8% of stage 3 AKI patients who required dialysis, as compared to 42.9% and 40.0% in stages 1 and 2, respectively. In the study by Rao et al. from the same city as our center, 28% of patients with AKI required dialysis, but none could receive it due to a lack of resources in the study setting [12]. In the cohort study done by Murdeshwar et al. on AKI in the PICU, 44% of children required dialysis. Only 14.5% of patients required dialysis in a study on AKI conducted by Krishnamurthy et al., with the majority (54.2%) receiving hemodialysis [2,8].

In this study, out of the total number of children enrolled (N=64), 24 (37.5%) of the participants had AKI that was completely resolved; 9 (14.1%) of the participants had partially resolved AKI; and 1 (1.6%) of the participants had unresolved AKI on discharge. Out of the total patients, 2 (3.1%) left against medical advice, and 28 (43.8%) of the participants died. Out of survivors at discharge (N=34), 61.76% (N=21) had complete renal recovery, and 35.29% (N=12) had partial renal recovery. In the study by Krishnamurthy et al. from 2013, mortality in children with AKI was 17.5% overall, which increased to 46.2% in patients with AKI in the PICU. In survivors, 82.5% had completely recovered, with 17.5% having partial renal recovery [2]. In a similar study by Rao et al. in Raipur, 87.4% of children with AKI in the PICU died as a consequence of AKI or coexisting MODS/shock [12].

Since the majority of the patients in our study required emergency and critical care (89.1% PICU stay), it was also associated with high mortality (43.8%), thus signifying the high level of sickness in most children. This mortality rate was comparable to that obtained by the study by Krishnamurthy et al. but was less than a similar study done by Rao et al. in Raipur in 2019 [2,12]. The mean PRISM3 score in patients who completely recovered from AKI was 9.43, as compared to patients who died, who had a mean PRISM3 score of 11.61. In the study by Krishnamurthy et al., PRISM III scores were higher in children dying than in survivors (31.2±14.1 vs. 16.6±12.3) [2]. The mean PRISM3 score for patients with stage 3 AKI was 10.5, which was the highest among all stages of AKI. A higher PRISM3 score had statistically significantly higher odds of mortality (OR = 1.14).

Out of 64 patients, 34 survived at discharge and were followed up after three months. Among them, three patients were lost to follow-up. Out of them, 19.35% (6/31) developed CKD. According to staging, stage 1 CKD was seen in two children at 33.33% (2/6), stage 2 was seen in three children at 50% (3/6), and stage 3 was seen at 16.66% (1/6). No child became dialysis-dependent. In the study on children by Murdeshwar et al., they studied the outcome of AKI patients at 90-day follow-up. 16% of children developed CKD (more than or equal to stage 2) [8]. Ashish et al. published a study in 2023 that showed that out of 215 children diagnosed with AKI, 24 died, and of the patients discharged, 10 developed CKD [6]. In a study from Pakistan conducted by S Bai et al., they found that of 130 children with AKI, 9 (6.9%) and 11 (8.5%) developed end-stage renal disease (ESRD) and CKD, respectively [14]. Hence, in our study, the development of CKD in AKI survivors was higher as compared to other studies.

Limitation of study

It was not possible to study the development of CKD in some patients after three months. Additional research should be conducted with extensive follow-up periods.

## Conclusions

We conclude that AKI incidence was high in critically ill children admitted to the PICU, even without primary renal disease. Mortality was also high in cases with AKI. Progression to CKD was also high so that long-term follow-up would have been better.
